# Effects of Intestinal FXR-Related Molecules on Intestinal Mucosal Barriers in Biliary Tract Obstruction

**DOI:** 10.3389/fphar.2022.906452

**Published:** 2022-06-13

**Authors:** Meng Yan, Li Hou, Yaoyao Cai, Hanfei Wang, Yujun Ma, Qiming Geng, Weiwei Jiang, Weibing Tang

**Affiliations:** ^1^ Department of Pediatric Surgery, Children’s Hospital of Nanjing Medical University, Nanjing, China; ^2^ Department of Pediatrics, Huai’an Maternal And Child Health Care center, Huai’an, China

**Keywords:** FXR, biliary atresia, intestinal microbiota, obeticholic acid, bile duct ligation

## Abstract

**Background:** The farnesoid X receptor (FXR) is a key factor regulating hepatic bile acid synthesis and enterohepatic circulation. Repression of bile acid synthesis by the FXR is a potential strategy for treating cholestatic liver disease. However, the role of intestinal FXR on the intestinal barrier and intestinal microbiota needs further investigation.

**Materials:** Intestinal tissues were collected from patients with biliary atresia or without hepatobiliary disease. Then, intestinal mRNA levels of FXR-related molecules were determined. To investigate the effect of FXR activation, bile-duct-ligation rats were treated with obeticholic acid [OCA (5 mg/kg/day)] or vehicle (0.5% methyl cellulose) per oral gavage for 14 days. The mRNA levels of intestinal FXR, SHP, TNF-α, FGF15 and bile acid transporter levels were determined. In addition, the intestinal permeability, morphologic changes, and composition of the intestinal microbiota were evaluated. Gut Microbiome was determined by 16S rDNA MiSeq sequencing, and functional profiling of microbial communities was predicted with BugBase and PICRUSt2. Finally, the role of OCA in injured intestinal epithelial cell apoptosis and proliferation was examined by pretreatment with lipopolysaccharide (LPS) in Caco-2 cells.

**Results:** The downstream of the FXR in ileum tissues was inhibited in biliary obstruction. Activation of the FXR signaling pathway by OCA significantly reduced liver fibrosis and intestinal inflammation, improved intestinal microbiota, and protected intestinal mucosa in BDL rats. OCA also altered the functional capacities of ileum microbiota in BDL rats. Significant differences existed between the controls and BDL rats, which were attenuated by OCA in the alpha diversity analysis. Principal coordinates analysis showed that microbial communities in BDL rats clustered separately from controls, and OCA treatment attenuated the distinction. Bugbase and PICRUSt2 analysis showed that OCA changed the composition and structure of the intestinal microbiota and improved the metabolic function of the intestinal microbiota by increasing the relative abundance of beneficial bacteria and reducing the relative abundance of harmful bacteria. Moreover, OCA reduced the apoptosis induced by LPS in Caco-2 cells.

**Conclusion:** The FXR agonist, OCA, activates the intestinal FXR signaling pathway and improves the composition and structure of the intestinal microbiota and intestinal barrier in BDL rats.

## Graphical Abstract

The FXR agonist, OCA, activates the intestinal FXR signaling pathway and improves the composition and structure of the intestinal microbiota and intestinal barrier in BDL rats.
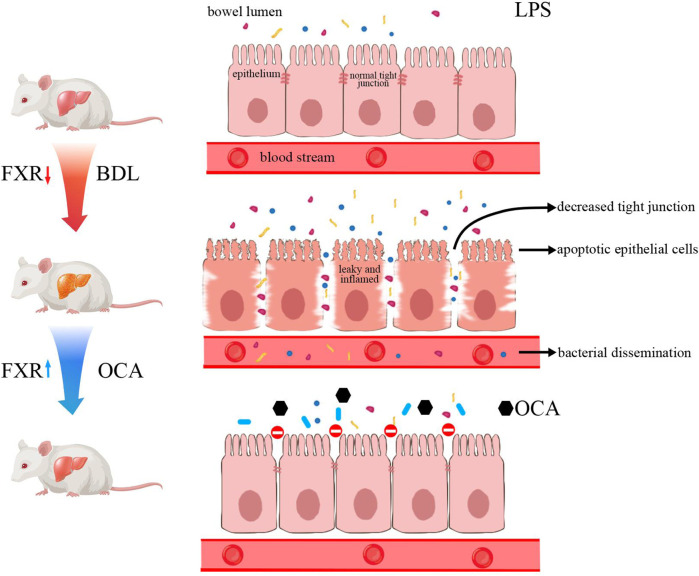

## Introduction

Biliary obstruction is a pathologic condition of intrahepatic cholestasis caused by partial or complete obstruction of the intra- and extra-hepatic bile ducts that restricts bile flow into the intestinal tract. Biliary atresia (BA) is the most common cause of biliary obstruction in newborns. The most common cause of BA is inflammation and fibrosis of extrahepatic bile ducts, which reduces the passage of bile into the intestine (Asai et al., 2015; Wang et al., 2020). The reduction of bile acids in the intestine results in apoptosis of intestinal epithelial cells and atrophy of the intestinal mucous (Assimakopoulos et al., 2004). When the intestinal mucosal barrier is injured, the intestinal bacteria and endotoxin enter the blood, resulting in bacteremia and endotoxemia, respectively (Sorribas et al., 2019).

Bile acids are produced from cholesterol through oxidation reactions in hepatocytes, then conjugated with glycine and taurine. The bile acid enterohepatic circulation is strictly regulated by a large number of enzymes and transporters, of which the most important regulatory factor is the farnesoid X receptor (FXR) (Gonzalez et al., 2017). Bile acids in the body are natural ligands of the FXR (Cariello et al., 2018), including the primary bile acids [chenodeoxycholic acid (CDCA) and cholic acid (CA)], the secondary bile acids [deoxycholic acid (DCA) and lithocholic acid (LCA)], and their conjugates with taurine and glycine (Makishima et al., 1999; Trabelsi et al., 2017). The FXR also plays an important role in the reabsorption of bile acids by regulating the bile acid transporter in the ileum (Trauner and Fuchs, 2022). Activation of the FXR pathway in the ileum influences expression of the apical sodium-BA transporter (ASBT), intestinal BA-binding protein (I-BABP), and organic solute and steroid transporter alpha-beta (OSTα/β), thus, increasing bile acid reabsorption into the blood (Dawson, 2011). In addition, intestinal FXR increases the expression of FGF19, a hormone secreted into the portal blood and transported to the liver to suppress CYP7A1 expression (Kong et al., 2012; Liu et al., 2020). Therefore, the FXR has an important effect on regulating bile acid synthesis and enterohepatic circulation.

The intestinal microbiota is closely related to bile acid metabolism via interaction with bile acids (Fiorucci and Distrutti, 2015; Schneider et al., 2018). Accumulation of bile acids in the liver leads to hepatocyte apoptosis and changes in the diversity of the intestinal microbiota (Wang et al., 2020). Bile acids and bacteria have a two-way regulation relationship; specifically, bile acids affect the survival and growth of bacteria, while bacteria regulate the consistency of intestinal bile acids (Stojancevic et al., 2012). The mechanism underlying bile acid toxicity on bacteria is multifactorial, and includes membrane effects, DNA damage, RNA structure changes, and protein denaturation (Assimakopoulos et al., 2007). Patients with BA do not have normal bile acids in the intestinal tract, which has a significant impact on the intestinal microbiota (Song et al., 2021). In mice, the intestinal microbiota not only regulates secondary bile acid metabolism but also regulates bile acid metabolism by reducing the level of T-βMCA, which improves intestinal FGF15 production and reduces BA synthesis (Sayin et al., 2013). Gut microbial dysbiosis results in endotoxin translocation into the portal vein, where activation of the NLRP3 inflammasome contributes to increased liver injury (Keitel et al., 2019). Besides, transferring the intestinal microbiota of cholestatic mice to healthy mice leads to severe liver damage (Kwong and Puri, 2021). Therefore, regulation of the intestinal microbiota is a potential treatment for liver disease secondary to BA.

The regulatory function of hepatic and intestinal FXR on bile acid metabolism has been extensively studied, but the role of intestinal FXR on the intestine has not been established. The activation of FXR has been proved to increase the expression of the tight-junction proteins claudin-1 and occluding in bacterial translocation (Verbeke et al., 2015). The present study determined the influence of the FXR agonist, obeticholic acid (OCA), in protecting the intestinal barrier in rats with obstructive jaundice by blocking the entry of bile into the intestine, excluding the indirect effect of bile on the intestine, then determining the effect of intestinal FXR on the intestine. We showed that OCA partly restored the enterohepatic circulation by increasing FGF19 expression. In addition, we demonstrated that the FXR has a protective effect on cholestatic liver injury and improves intestinal epithelial cell apoptosis in BDL rats, thus providing a new potential target for clinical prevention of intestinal mucosal barrier injury in patients with obstructive jaundice.

## Materials and Methods

### Tissues and Gut Microbial Collection

The current study involved 24 infants without hepatobiliary disease and 16 BA patients at the Children’s Hospital of Nanjing Medical University from November 2017 to December 2020. All BA patients were diagnosed based on intraoperative cholangiograms and pathologic evaluation of liver biopsies at the Children’s Hospital of Nanjing Medical University. Tissue samples were collected from BA patients undergoing the Kasai procedure. Matched controls were derived from patients without liver failure or malignancies, and were confirmed to not have BA or other congenital malformations. No study subjects were recently treated with antibiotics. All tissue samples were immediately frozen in liquid nitrogen and stored at −80°C. We acquired written informed consent from the subjects or their legal guardians. Ethics approval was given by the Research Ethics Committee of the Children’s Hospital of Nanjing Medical University.

### Animals Experiment Design

Animals were randomized into three groups and fed a regular diet. For the bile duct ligation (BDL) model, rats were anesthetized with chloral hydrate, then the bile duct was exposed and ligated with two non-resorbable surgical sutures. Rats that underwent BDL were divided into two groups (*n* = 6–7). After the procedure, rats received the treatment with vehicle (0.5% methylcellulose) or OCA (5 mg/kg/day dissolved in 0.5% methylcellulose) by daily gavage for 2 weeks. Two weeks later, all animals were sacrificed under anesthesia. Ileum content, the ileum, serum, and fecal samples were collected and immediately stored at −80°C.

### Serum Measurements

Alanine aminotransferase (ALT) and aspartate aminotransferase (AST) were measured using an automated bioanalyzer (Thermo Scientific Indiko Plus, Wuhan, China). The serum lipopolysaccharide (LPS) levels were determined using enzyme-linked immunosorbent assay (ELISA) kits (RayBiotech, Inc., United States).

### Intestinal Gene Expression

Ileum tissues (3–5 cm) were collected at 0.5–1.5 cm proximal to the ileocecal flap for gene expression studies. The process of tissue homogenates, RNA extraction and real-time quantitative polymerase chain reaction followed the protocol. The primer sequence is shown in Table 1, 2.

**TABLE 1:** Primers for qRT-PCR evaluation of gene expression levels in human.

	Human primer sequence (5’ to 3’)
*FXR*	F: GGG​ACA​GAA​CCT​GGA​AGT​GG
	R:GCCAACATTCCCATCTCTTTGC
*GAPDH*	F: GCA​CCG​TCA​AGG​CTG​AGA​AC
	R: GGA​TCT​CGC​TCC​TGG​AAG​ATG
*SHP*	F: CTC​ACT​GGG​TGC​TGT​GTG​AA
	R: AAG​AAG​GCC​AGC​GAT​GTC​AA
*TNF-α*	F : GATCGGTCCCAACAAGGAGG
	R : GCTTGGTGGTTTGCTACGAC
*FGF19*	F : TGTGTGGTGGTCCACGTATG
	R : CGGATCTCCTCCTCGAAAGC

**TABLE 2:** Primers for qRT-PCR evaluation of gene expression levels in rats.

	Rat primer sequence (5’ to 3’)
*fxr*	F: GGG​ACA​GAA​CCT​GGA​AGT​GG
	R: GCC​AAC​ATT​CCC​ATC​TCT​TTG​C
*gapdh*	F: GCA​CCG​TCA​AGG​CTG​AGA​AC
	R: GGA​TCT​CGC​TCC​TGG​AAG​ATG
*shp*	F: CTC​ACT​GGG​TGC​TGT​GTG​AA
	R: AAG​AAG​GCC​AGC​GAT​GTC​AA
*tnf-α*	F: GAT​CGG​TCC​CAA​CAA​GGA​GG
	R: GCT​TGG​TGG​TTT​GCT​ACG​AC
*fgf15*	F: TGT​GTG​GTG​GTC​CAC​GTA​TG
	R: CGG​ATC​TCC​TCC​TCG​AAA​GC
*i-babp*	F: TATGGCCTTCACCGGCAA
	R: TAC​GTC​CCC​TTT​CAA​TCA​CA
*ostα*	F: GGG​CAG​ATC​GCT​TGC​TCA​CC
	R: TCA​GGC​TTT​GAG​CGT​TGA​GT
*asbt*	F: TGG​GTT​TCT​TCC​TGG​CTA​GAC​T
	R: TGT​TCT​GCA​TTC​CAG​TTT​CCA​A

### Western Blot Analysis

The frozen tissues and cells were lysed on ice with RIPA buffer (Solarbio). The expression levels of target proteins in the ileum were detected by respective primary antibodies. For protein density, each band was determined and then quantified by using Image J software. Primary antibodies involved in this study includes: anti-cleaved caspase-3 (1:2000, Cell Signaling Technology), anti-PCNA (1:1,000, Santa Cruz), anti-β-actin (1:1,000, Santa Cruz).

### Gut Microbiota Sequencing and Microbial Analysis

Genomic DNA was extracted from fecal samples using a E. Z.N.A. ®Stool DNA Kit (D4015, Omega, Inc., United States) according to manufacturer’s instructions. Amplicons of the V3–V4 region of the 16S rDNA gene were conducted by using a 341F/805R primer pair. Sequencing was carried out on a NovaSeq PE250 platform. Sequence data analyses were mainly conductd by using Quantitative Insights Into Microbial Ecology2 (QIIME2) and R packages (v3.5.2). For dereplication, feature table and feature sequence were obtained by DADA2. The raw data of 16Sr DNA gene sequencing and metabolomic sequencing quality control in each sample are provided in the supplementary materials. Bugbase was used for the predictions of the functional profile of a microbial community based on 16S rDNA sequence data. Based on Kyoto Encyclopedia of Genes and Genomes (KEGG) functional pathways, PICRUSt2 was used to analyze the metabolic networks of ileum microbiota, with an emphasis on the enriched pathways.

### Cell Culture and Treatments

The human intestinal epithelial cell line, Caco-2, was cultured in RPMI-1640 medium supplemented with 10% fetal bovine serum (FBS), 0.1 mg/ml of streptomycin, 100 U/mL of penicillin, and 2 mmol/L L-glutamine. Caco-2 cells were incubated in a humid atmosphere (5% CO_2_ and 95% air) at 37°C. Caco-2 cells were treated with LPS (100 μg/ml) for 24 h to induce intestinal epithelial injury followed with the FXR agonists, OCA (10 μM) an additional 24 h.

### Cell Viability Assay

Cell viability was detected with a CCK8 kit (Beyotime, Nantong, China). After the above treatment, cells were cultivated in 96-well microplates at a concentration of 1 cells/well × 10^4^ cells/well and incubated in medium containing 5% FBS for 12 h. CCK-8 (10 μl/well) was then added to the wells and 96-well microplates were incubated at 37°C for 1–4 h. The absorbance at 450 nm was measured using a Tecan Infinite M200 multimode microplate reader (Tecan, Mechelen, Belgium).

### Cell Apoptosis Analysis

The TdT-mediated dUTP nick-end labeling (TUNEL) assay was used in rat ileum sections to measure apoptosis of intestinal epithelial cells according to the manufacturer’s instructions (Beyotime, Shanghai, China). To detect Caco-2 cell apoptosis, cells were harvested and stained with the Annexin V-FITC/propidium iodide kit (KeyGen Biotech, Nanjing, China) following the manufacturer’s instructions. Apoptosis rates were analyzed using FlowJo V7 software.

### Statistical Analysis

All statistical analyses are conducted using GraphPad Prism (version 8; GraphPad Software Inc., San Diego, CA, United States). Data are presented as the mean ± SEM. Statistical comparisons were made using the one-way analysis of variance (ANOVA) with tukey’s post hoc test or double-sided Student’s t-test where appropriate. *p* value < 0.05 was considered significant. The qualitative data represent three independent experiments.

## Results

### Clinical Information Analysis

Clinical information, including age, gender, and biochemical indicators, was obtained from 16 BA patients and 24 healthy controls. The mean ages of BA patients and controls were 55.63 ± 6.11 and 70.81 ± 20.25 days, respectively; there was no significant difference in mean age between the BA patients and healthy controls (Table 3). The serum alanine aminotransferase (ALT), aspartate aminotransferase (AST), alkaline phosphatase (ALP) direct bilirubin (DBIL), and total bilirubin (TBIL) levels were significantly increased in the BA group (Table 4).

**TABLE 3:** Demographic clinical features of study subjects.

Information	Control (*n* = 24)	Biliary Atresia (*n* = 16)	*p* t
Age (days, mean SE)	70.81 (20.25)	55.63 (6.11)	0.5535 0.5979
Male (%)	15 (62.5)	8 (50)	
Female (%)	9 (37.5)	8 (50)	

**TABLE 4:** Liver function.

Indicator	Control (*n* = 24)	Biliary Atresia (*n* = 16)	*p*	t
ALT (U/L)	35.25 ± 4.416	161.2 ± 18.34	<0.0001	7.579
AST (U/L)	40.75 ± 2.837	245.5 ± 34.61	<0.0001	7.020
ALP(U/L)	320 ± 21.06	577.6 ± 45.22	<0.0001	5.599
DBIL (μmol/L)	2.068 ± 0.1772	131.8 ± 8.741	<0.0001	17.21
TBIL (μmol/L)	5.599 ± 0.4707	167.6 ± 11.43	<0.0001	16.41

### The Farnesoid X Receptor-FGF19 Signaling Pathway and Intestinal Barrier Are Impaired in Patients With Biliary Atresia

**FIGURE 1:**
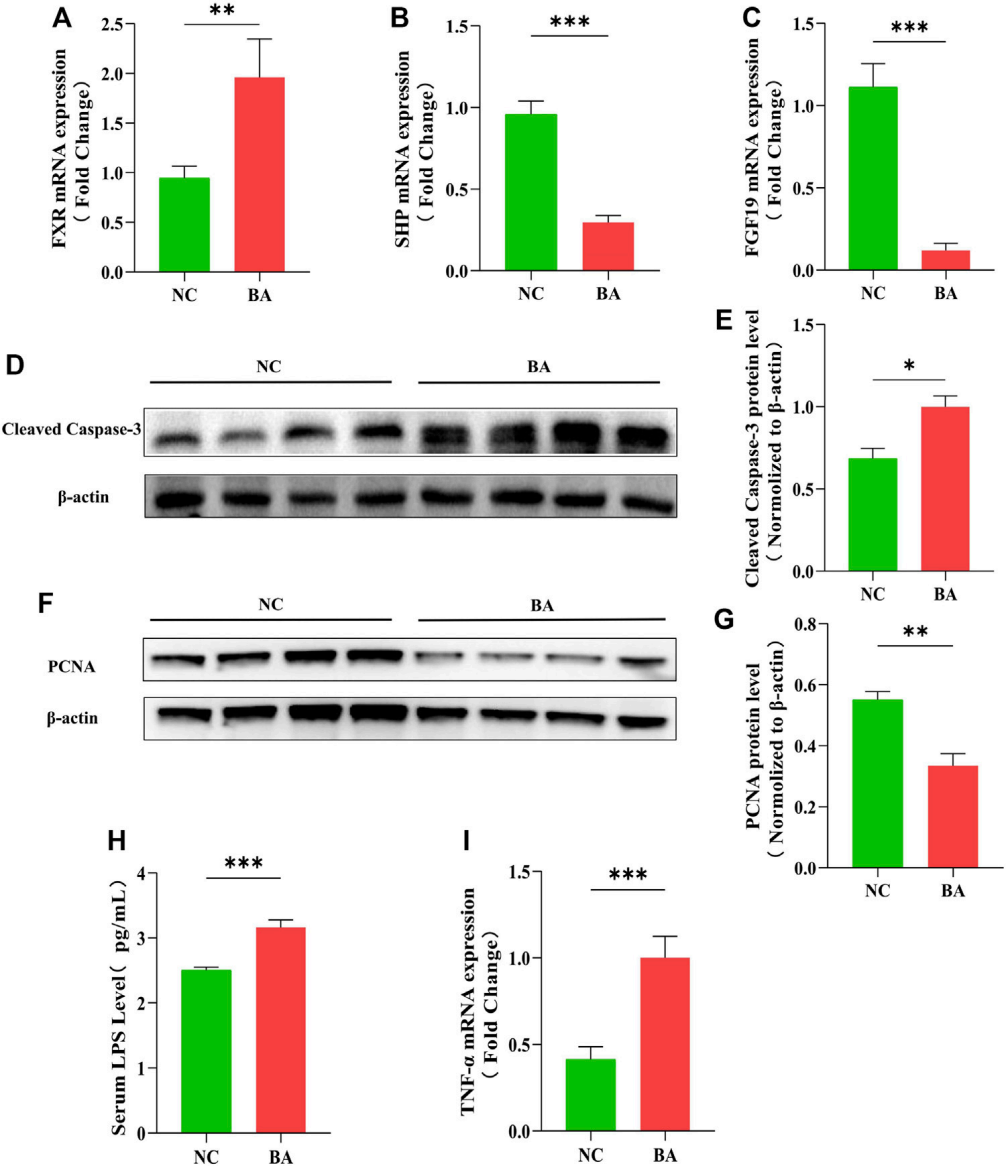
The FXR-FGF19 signaling pathway and intestinal barrier are impaired in patients with biliary atresia. **(A–C)** The expression of intestinal FXR, SHP, and FGF19 mRNA in BA tissues (*n* = 16) and controls (*n* = 24). **(D–G)** The levels of PCNA and cleaved caspase-3 protein expression in BA tissues and controls. **(H)** Serum LPS detected by ELISA. **(I)** Intestinal expression of TNF-α mRNA. The data are expressed as the mean ± SEM. (*p < 0.05, **p < 0.01, ***p < 0.001).

First, FXR-related molecule expression in the 16 BA and 24 control samples was determined. The FXR was highly expressed in BA patients, while the small heterodimer partner (SHP) and FGF19 were significantly lower than in the control group (Figures 1A–C). The FXR-FGF19 signaling pathway was damaged in biliary obstruction, which might be involved in the development of BA. We also evaluated the apoptosis, proliferation and inflammatory response in intestinal tissues. The protein level of cleaved caspase-3 was greatly increased in BA samples (Figures 1D,E). Meanwhile, the expression levels of PCNA was significantly decreased in BA (Figures 1F,G). Serum LPS and intestinal TNF-α were significantly increased in BA patients (Figures 1H,I).

### Obeticholic Acid Modulated Farnesoid X Receptor Signaling Pathway and Attenuated Intestinal Injury in Bile Duct Ligation Rats

**FIGURE 2:**
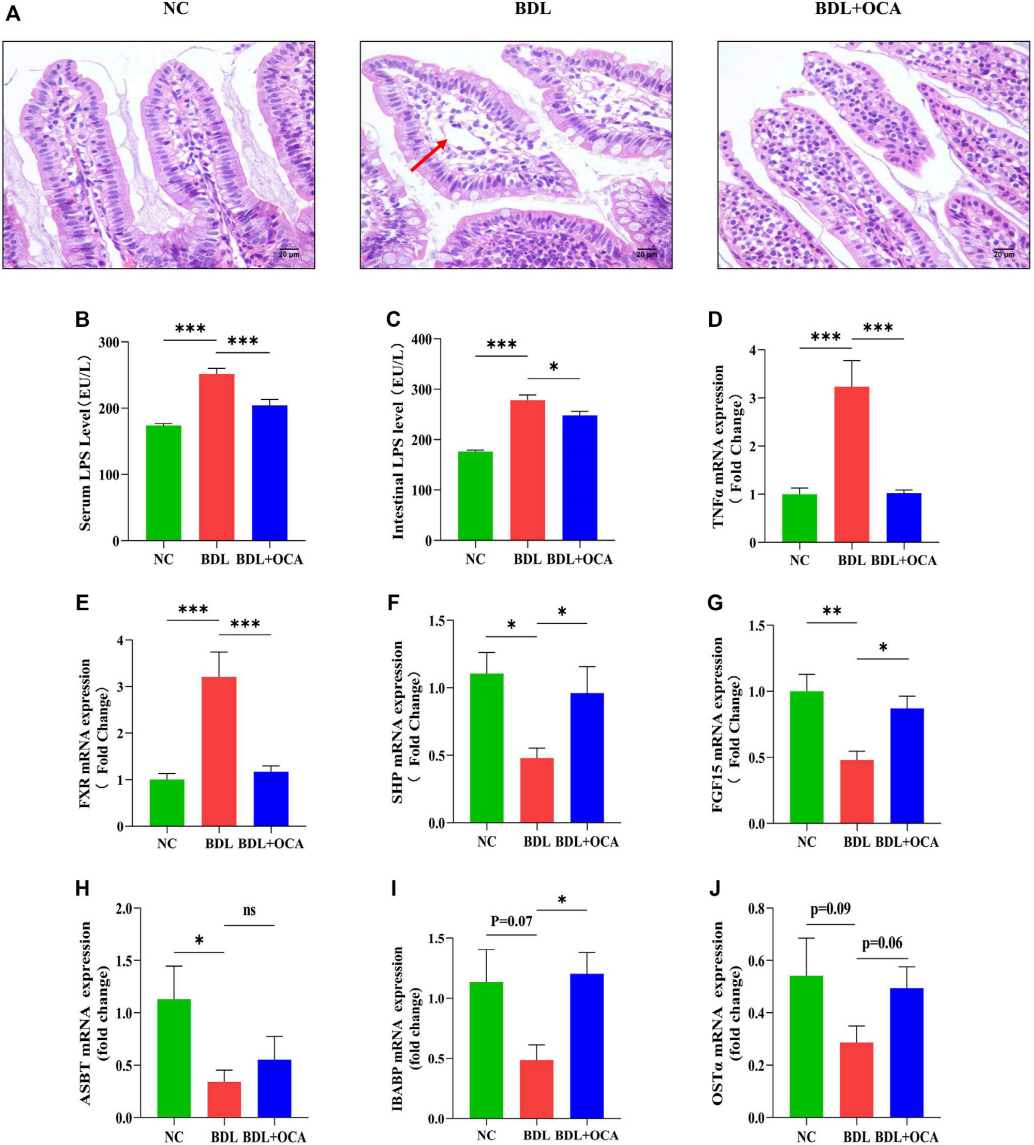
OCA modulates the FXR signaling pathway and attenuates intestinal injury in BDL rats. **(A)** Representative images of intestinal sections stained with hematoxylin and eosin. **(B,C)** Serum and intestinal content of LPS detected by ELISA. **(D)** Intestinal mRNA expression of TNF-α. **(E–G)** Ileum expression of FXR, SHP, and FGF15 mRNA. **(H–J)** The bile acid transporter mRNA expression in ileum. The data are expressed as the mean ± SEM (*n* = 7–9). (OCA [5 mg/kg/day]) (*p < 0.05, **p < 0.01, ***p < 0.001)

To gain further insight into the effect of OCA, we determined the expression of FXR-related molecules and the impact on BDL rat intestines. HE staining revealed impaired intestinal mucosal architecture in BDL rats, in which intestinal villi were short, thick, and edematous, as shown by the red arrow. The mucosal injury was significantly decreased in BDL rats treated with OCA (Figure 2A). The serum and intestinal level of LPS were significantly increased in BDL rats compared with the control group, whereas OCA caused a marked decrease in the serum levels of these markers (Figures 2B,C). Intestinal TNF-α was also significantly increased in BDL rats, which was reduced by OCA (Figure 2D). Then, we examined the ileum FXR signaling pathway in the BDL rats and the changes produced by OCA. Expression of FXR in BDL rats ileum was higher than controls (Figure 2E). However, the reduced activity of the FXR signaling pathway was indicated by the decreased ileum expression of the FXR target gene, such as SHP and FGF15. OCA effectively modulated the ileum FXR signaling pathway in BDL rats (Figures 2F,G). Furthermore, lack of bile acid in ileum contributde to the decreased trend of bile acid transporter mRNA expression, and FXR activation partially reversed this change (Figures 2H–J).

OCA attenuated BDL-induced apoptosis of ileum epithelial cells.

**FIGURE 3:**
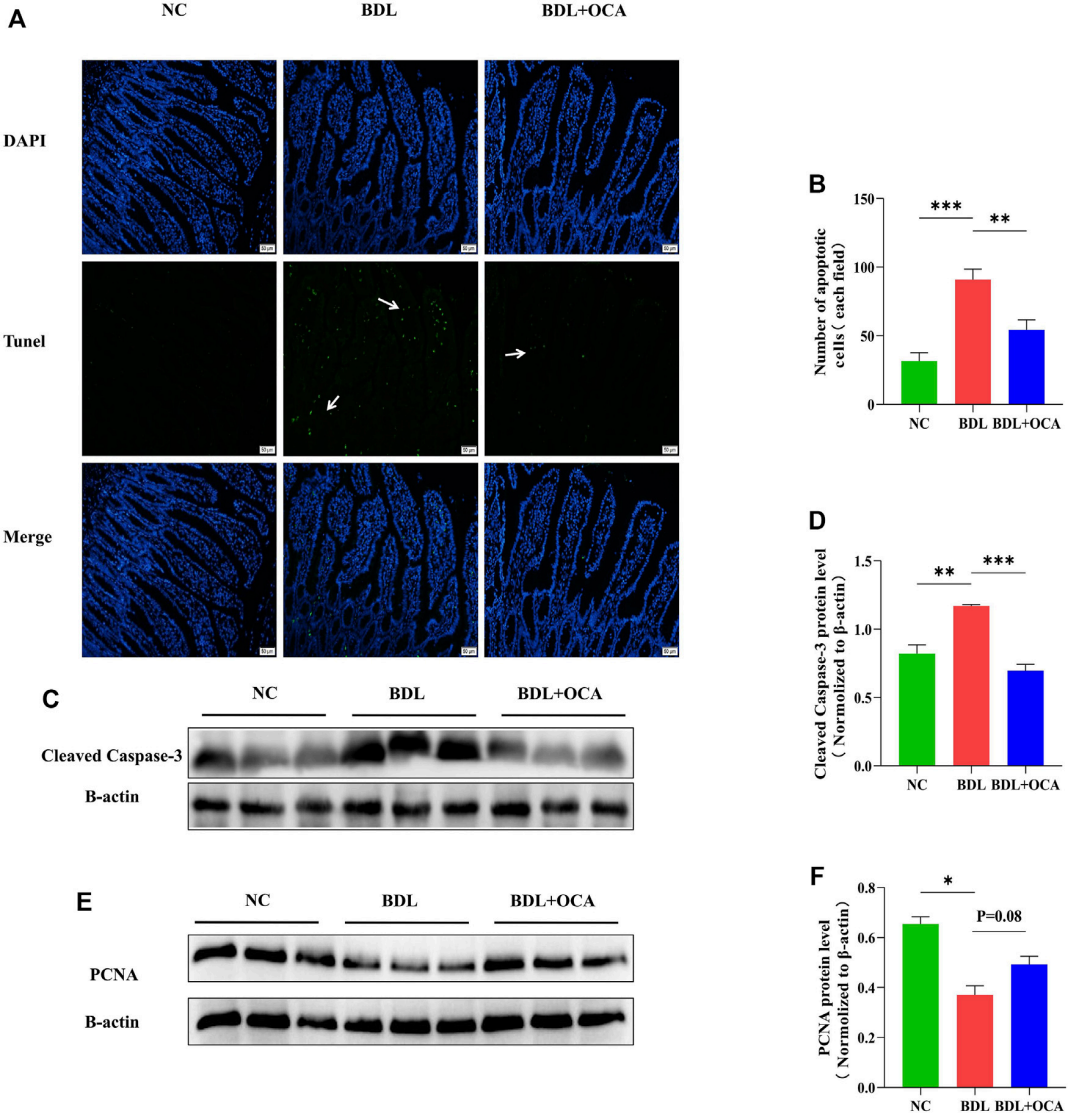
OCA attenuates BDL-induced apoptosis of ileum epithelial cells. **(A)** TUNEL assay of small ileum sections. **(B)** The number of TUNEL-positive cells in the intestinal epithelium is shown. **(C,D)** Western blots of cleaved caspase-3 in ileum tissues from rats. **(E,F)** The protein expression level of PCNA in three groups. Bar graphs represent the mean ± SEM (*n* = 7–9). (OCA [5 mg/kg/day]) (*p < 0.05, **p < 0.01, ***p < 0.001)

Previous studies have shown that bile acids stimulate intestinal epithelial proliferation (Yasuda et al., 2007). A lack of bile acid in the intestinal lumen is mainly attributed to impaired cell proliferation and increased apoptosis (Assimakopoulos et al., 2007). To determine whether the OCA-mediated protective effect against BDL-induced disruption of intestinal barrier function was due to an inhibition of cell apoptosis, we performed the TUNEL assay in ileum sections. Compared with controls, intestinal epithelial cell apoptosis was significantly increased in BDL rats, as shown by the white arrow (Figure 3A). The number of apoptotic cells was clearly greater than controls (Figure 3B). As expected, intestinal epithelial cell apoptosis was markedly inhibited by OCA. Similar results were observed in a Western blot analysis, the levels of cleaved caspase-3 protein detected by western blot were increased in BDL rats compared with controls, and OCA attenuated BDL-induced upregulation of cleaved caspase-3 (Figures 3C,D). Consistent with these results, Treatment with OCA increased the expression of the PCNA in BDL rats (Figures 3E,F).

### Obeticholic Acid Ameliorated the Dysbiosis of the Ileum Microbiota in Bile Duct Ligation Rats

**FIGURE 4:**
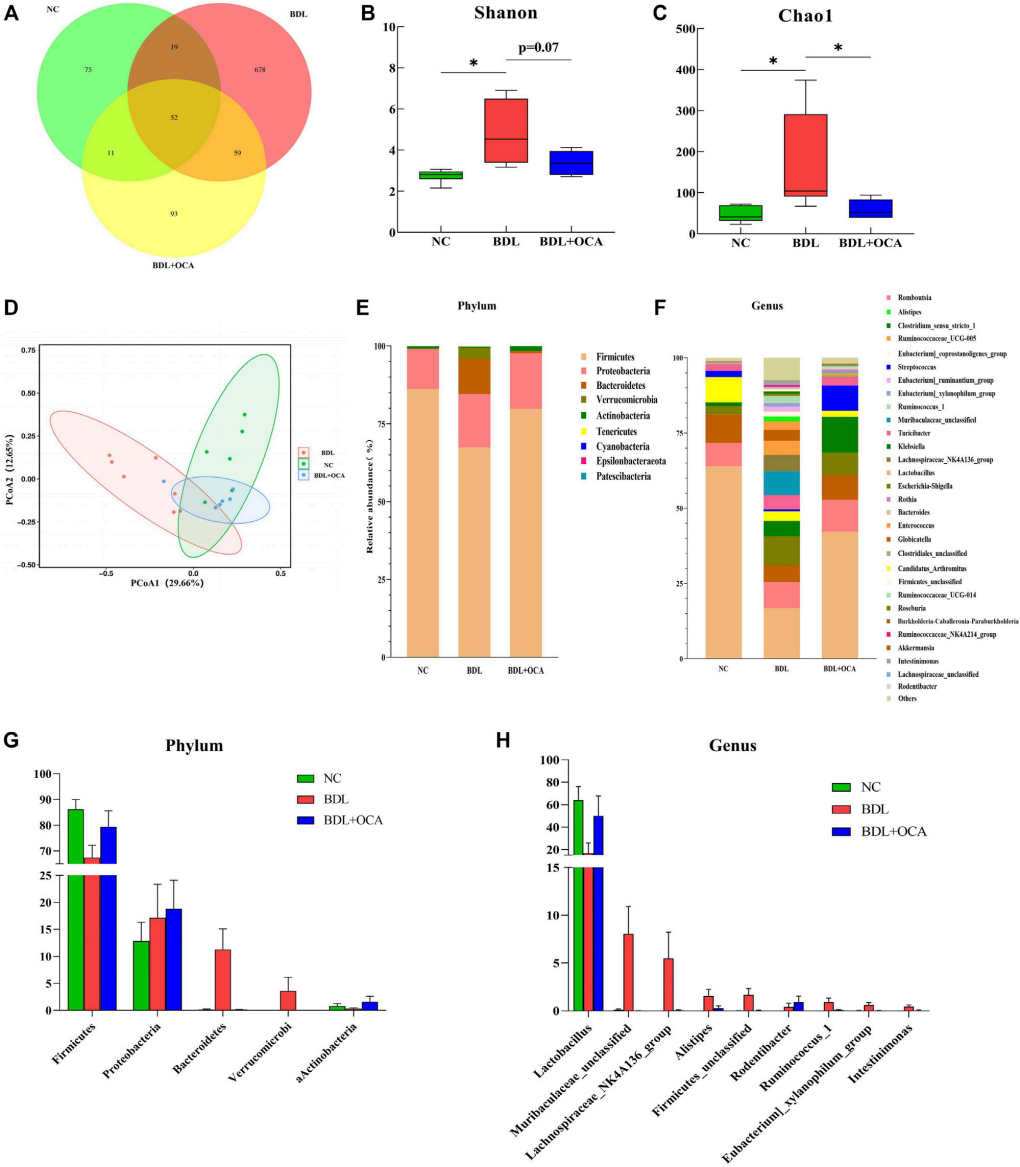
OCA ameliorates dysbiosis of the ileum microbiota in BDL rats. **(A)** Venn diagram showing exclusive features per group. **(B,C)** Alpha-diversity analysis of ileum microbiota (Shannon and Chao1 index). **(D)** Differences of ileum microbial β-diversity. Beta-diversity was analyzed by principal coordinates using weighted Unifrac distances. **(E,F)** Composition of ileum microbiota at the phylum and genus levels. **(G,H)** Bacterial phyla and genera in ileum microbiota that were significantly changed in three groups and corresponding relative abundance. The data are expressed as the mean ± SEM (*n* = 6–7). (OCA [5 mg/kg/day]) (*p < 0.05, **p < 0.01, ***p < 0.001).

To determine the mechanism involved in OCA-mediated protection against BDL-induced intestinal injury, we assessed the effects of BDL and OCA on microbiota composition in the ileum by amplifying and analyzing amplicons from the V3-V4 region of the 16S rDNA gene. A Venn diagram showed 75 features in the controls, 678 features in BDL rats, and 93 features in BDL rats treated with OCA (Figure 4A). The Shannon and Chao1 index was calculated to assess α-diversity in bacterial diversity. Significant differences existed between the controls and BDL rats, which were attenuated by OCA (Figures 4B,C). Principal coordinates analysis showed that microbial communities in BDL rats clustered separately from controls, and OCA treatment attenuated the distinction (Figure 4D). Differences in the microbiota community composition, ordered by relative abundance in the samples, were observed at the phylum and genus levels (Figures 4E,F).

As shown in Figure 4G, BDL rats had a reduced relative abundance of phylum Firmicutes that harbor bacteria with high bile salt hydrolase activity, which was increased by OCA treatment (Jones et al., 2008). In contrast, Bacteroidetes, a major bacterial phylum harboring bacteria with low bile salt hydrolase (BSH) activity, was significantly increased in BDL rats and attenuated by OCA treatment. At the genus level, the abundance of *Lactobacillus* was significantly decreased, and the abundance of the Lachnospiraceae NK4A136 group, Alistipes, Eubacterium_ruminantium_group, Intestinimonas, Ruminococcus_1, and Ruminococcaceae_NK4A214_group was significantly increased in BDL rats; however, OCA changed the unique enteric microbiome of BDL rats (Figure 4H). Compared with BDL rats, OCA-treated rats had fewer Lachnospiraceae_NK4A136_group, Eubacterium_ruminantium_group, Ruminococcus_1, and Intestinimonas, and more abundance of *Lactobacillus*.

### Obeticholic Acid Altered the Functional Capacities of Ileum Microbiota in Bile Duct Ligation Rats

**FIGURE 5:**
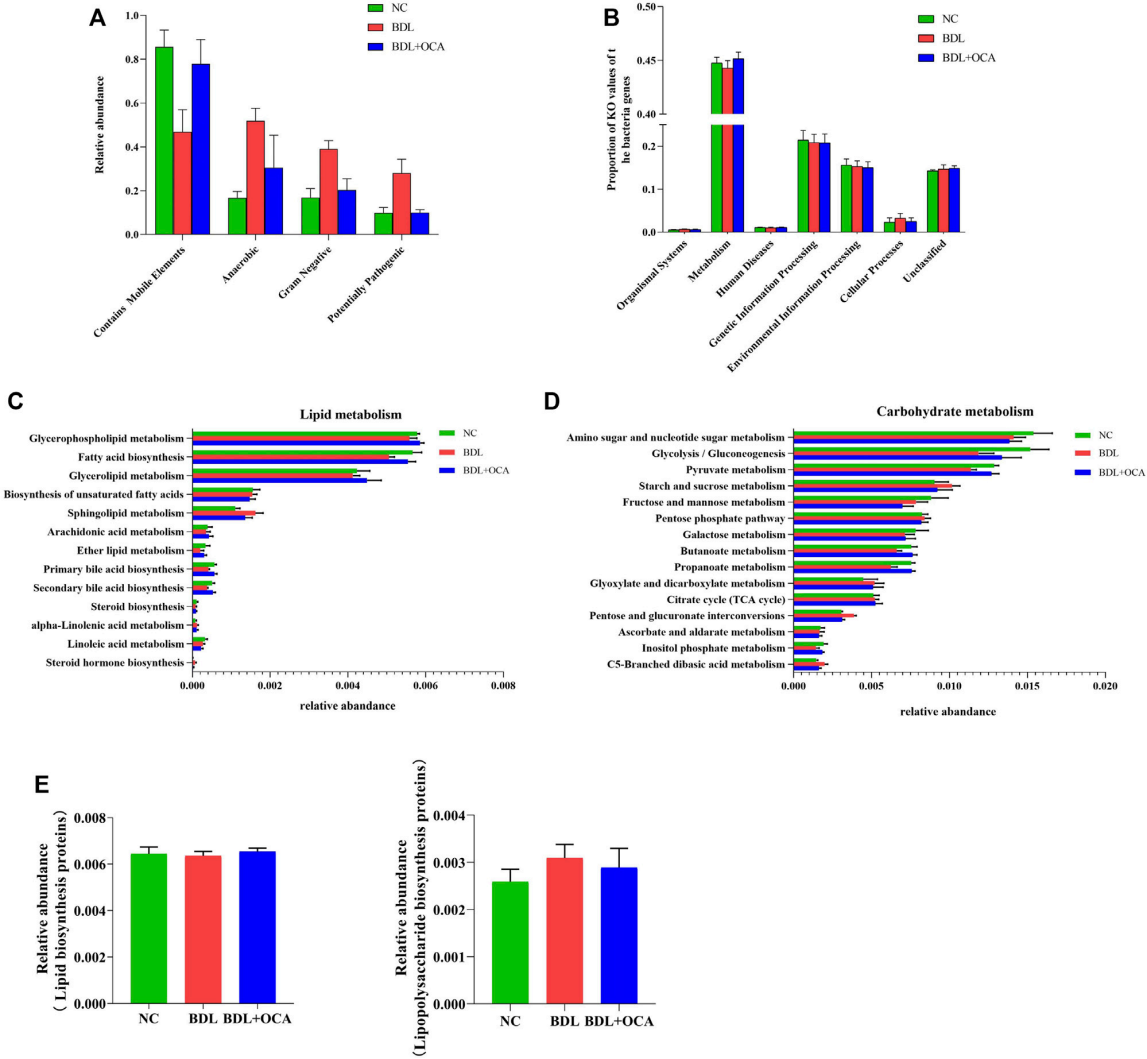
Obeticholic acid ameliorated the dysbiosis of the ileum microbiota in bile duct ligation rats. **(A)** Relative abundances of four potential phenotypes predicted by BugBase. **(B)** Predicted functional profiles in all groups. **(C,D)** Relative abundance of the subordinate pathways of lipid and carbohydrate metabolism. **(E)** Comparison of gene sets associated with lipopolysaccharide biosynthesis and lipopolysaccharide biosynthesis proteins. The data are expressed as the mean ± SEM (*n* = 6–7). [OCA (5 mg/kg/day)].

Four potential phenotypes (anaerobes, containing mobile elements, gram-negatives, and potentially pathogenic) were predicted to be significantly different in the three groups using BugBase. At the microbial community level, gene functions related to anaerobes, gram-negatives, and potentially pathogenic were increased in BDL rats. OCA treatment reduced the enrichment of gram-negatives and potentially pathogenic, possibly attributed to a decrease in the abundance of Bacteroidetes (Figure 5A). In addition, we gained functional prediction of the fecal bacteria through PICRUSt2 based on a KEGG database. The primary identified pathway of ileum microbiota was related to metabolism (Figure 5B), which was significantly decreased in BDL rats; however, OCA treatment improved enrichment of metabolism compared to BDL rats.

Furthermore, the relative abundances of ileum microbiota involved in primary and secondary bile acid biosynthesis-associated lipid metabolism were lower in BDL rats than controls, which may have been associated with lower bile acid levels (Figure 5C). Compared with BDL rats, OCA-treated BDL rats had an increased abundance of primary and secondary bile acid biosynthesis. The relative abundances of ileum microbiota related to sphingolipid metabolism and steroid hormone biosynthesis, which are involved in lipid metabolism, and were more significant in BDL rats than in other groups (Figure 5C). Subordinate pathways of carbohydrate metabolism were also analyzed and compared. Pyruvate metabolism, glycolysis/gluconeogenesis, an amino sugar, and nucleotide sugar metabolism were enriched in three groups of ileum microbiota (Figure 5D); however, there was an insignificant difference between the three cognitive groups in lipopolysaccharide biosynthesis and lipopolysaccharide biosynthesis proteins (Figure 5E).

### Obeticholic Acid Protected Against Lipopolysaccharide-Induced Apoptosis *in Vitro*

**FIGURE 6:**
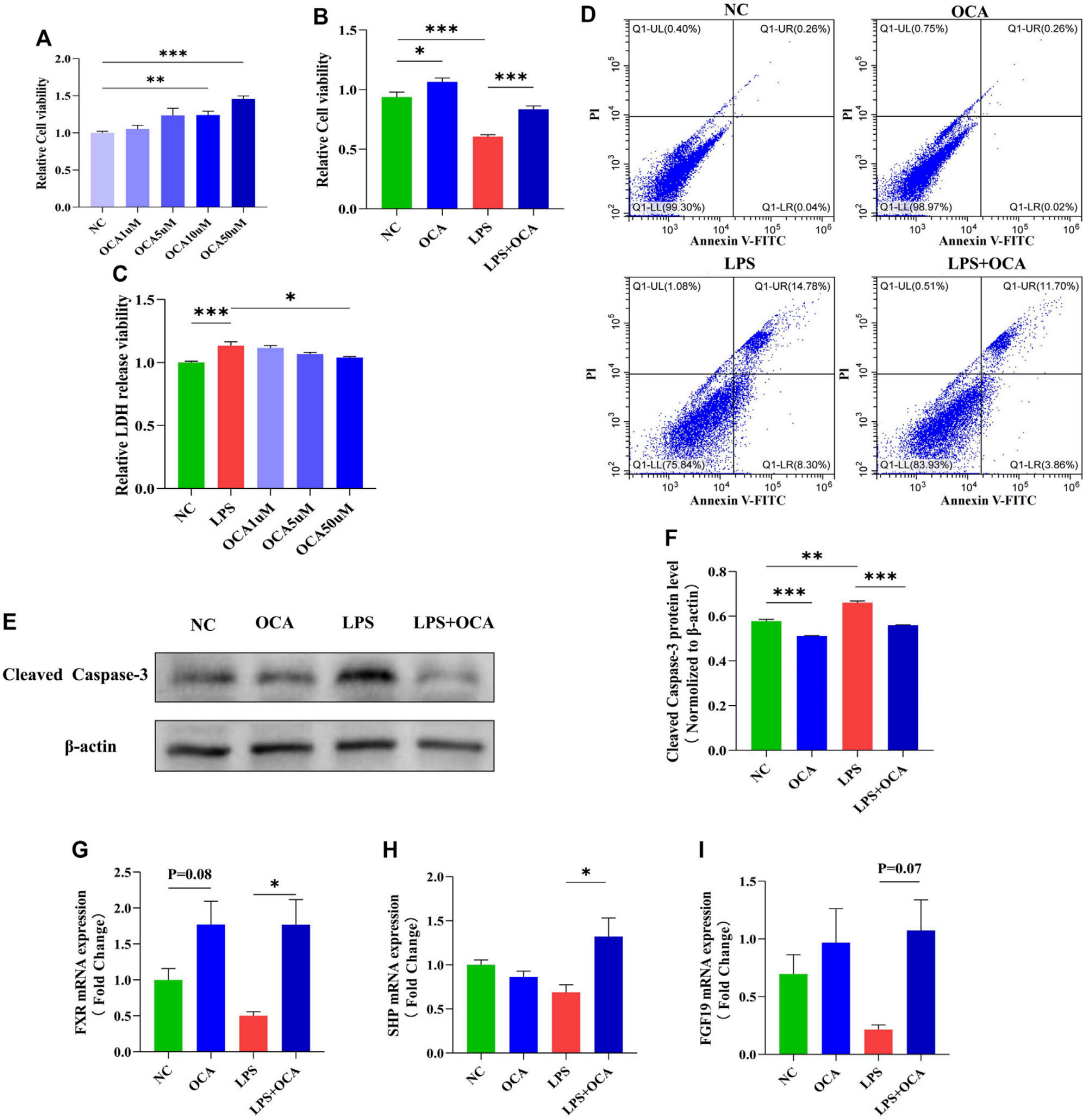
OCA protected against LPS-induced apoptosis *in vitro*. **(A,B)** cell viability evaluated by CCK8 in the Caco-2 cell line. **(C)** LDH released in the supernatant of cultured cells was measured at the indicated time. **(D)** Cell apoptosis assay detected by flow cytometry in the Caco-2 cell line. **(E,F)** The level of cleaved caspase-3 protein expression in the Caco-2 cell line. **(G–I)** Real-time PCR analysis of FXR and its target genes FGF-19 and SHP. (OCA [10 μM], LPS [100 μg/ml]) (*p < 0.05, **p < 0.01, ***p < 0.001)

The presence of bile acid in the intestine contributes to a normal gut barrier function by promoting intestinal epithelial cell proliferation. In biliary obstruction, intestinal mucosal injury is mainly due to more LPS caused by intestinal flora disorder (Assimakopoulos et al., 2007; Luo et al., 2022). To further confirm the effect of OCA on the proliferation and apoptosis of intestinal mucosal epithelial cells, Caco2 cells were incubated with LPS and OCA *in vitro*. OCA promoted the proliferation of Caco-2 cells based on concentration (Figure 6A). OCA improved the inhibitory effect of LPS on cell viability and cell proliferation rates (Figure 6B). OCA gradually reduced the released LDH in Caco-2 cells incubated with LPS as the OCA concentration increased (Figure 6C). Next, treatment of OCA significantly prevented apoptosis of Caco-2 cells incubated with LPS (Figure 6D-F). In addition, we determined whether OCA directly activates FXR by RT-PCR. OCA restored LPS-suppressed FXR expression in Caco-2 cells (Figures 6G–I).

## Discussion

Because biliary obstruction not only causes inflammation and fibrosis of the liver, biliary obstruction can also cause damage to the intestinal barrier, leading to endotoxemia. Therefore, protecting the intestinal mucosal barrier during biliary obstruction is an important treatment strategy. Indeed, our study showed that intestinal permeability, inflammation, and intestinal epithelial apoptosis was increased during biliary obstruction.

It is well-accepted that the FXR regulates the synthesis, transport, and enterohepatic circulation of bile acids by modulating the expression of related genes in the liver and small intestine (Liu et al., 2020). The main finding of this study showed that the relationship between the FXR pathway and the intestinal mucosal barrier during biliary obstruction. In BDL rats, the intestinal FXR signaling pathway was inhibited after bile duct ligation, and the restoration of the FXR pathway improved intestinal barrier damage and intestinal microbiota imbalance. This finding provides new insight into the role of the FXR pathway in intestinal barrier injury.

Protection of the activated FXR pathway on intestinal barrier integrity was associated with reduced liver fibrogenesis. Hepatic accumulation of bile acid plays a pivotal role in the apoptosis of hepatocytes and bile duct hyperplasia, and eventually leads to liver fibrosis and cirrhosis in biliary obstruction (Fickert and Wagner, 2017). The decrease in intestinal bile leads to a deficiency in intestinal FXR activity, which weakens the inhibitory effect of CYP7A1 and leads to increased bile acid production and aggravation of liver fibrosis (Liu et al., 2020). In agreement with previous studies (Liu et al., 2020; Tsai et al., 2020; Modica et al., 2012), our results showed that OCA reduced liver fibrosis in rats with cholestasis by BDL (Supplementary Figure S1). The mechanisms underlying improved hepatic fibrosis by OCA remain unclear, although there are several explanations. Previous research has shown that bile acid activates intestinal FXR and elevates FGF15/19, which binds the FGF receptor and the β-Klotho complex on the surface of hepatocytes to inhibit CYP7A1 expression (Hartmann et al., 2018; Lee et al., 2018). In our study, biliary obstruction reduced intestinal bile acid concentration, leading to a significant reduction of SHP and FGF15 expression. Moreover, OCA significantly promoted the expression of SHP and FGF15, which inhibited CYP7A1 expression and decreased the production of bile acids. In addition, mitigation of liver fibrosis caused by OCA may be achieved by regulating the intestinal microbiota and restoring the intestinal barrier, thereby improving the “gut-liver axis” circulation, reducing liver inflammation, and ultimately alleviating liver fibrosis. Previous studies have shown that antibiotics which improve liver function in cholestatic patients indicate an active role of the microbiome in mediating liver injury during cholestasis (Tabibian et al., 2013). In previous study, decreased LPS production from the gut by OCA treatment activated TLR-4 and TLR-9, thus promoting inflammation, steatosis, and fibrosis, which might have contributed to reduced liver fibrosis (Lichtman et al., 1990).

The presence of bile in the intestine promotes intestinal epithelial cell proliferation and protects against apoptotic cell death, which contributes to the integrity of the intestinal barrier (Keitel and Häussinger, 2011). With the absence or reduction of bile in the intestine, the intestinal mucosal barrier is damaged, and the intestinal permeability is increased so that pathogenic bacteria and LPS translocate into the blood, causing bacteremia and endotoxemia (Iida et al., 2009; Wang et al., 2010). More LPS production facilitates liver and intestinal mucosal barrier damage through the LPS-TLR4 signaling pathway (An et al., 2022). Activation of the FXR pathway or the FXR agonists protects the mucosal integrity by regulating the expression of downstream genes related to mucosa protection and defense against inflammation in rodents (Vavassori et al., 2009; Gadaleta et al., 2011). Interestingly, in the case of bile duct obstruction, adding OCA can activate the FXR like bile acid, promoting the proliferation of intestinal epithelial cells and preventing cell apoptosis. Moreover, similar results were demonstrated *in vitro*, in which OCA attenuated LPS-induced apoptosis of Caco-2 cells. In the other hand, the increase of LPS disruptes bile acid metabolism (Xiong et al., 2017; Zhang et al., 2020), and contributes to the damage of liver and intestinal barrier. In the study of Sai Wang et al., LPS inhibited the expression of FXR signaling pathway in mouse primary hepatocytes (Wang et al., 2022), and we also found that OCA actived the FXR signaling pathway in lps-incubated Caco-2 cells, which may be involved in the mechanisms affecting the proliferation and apoptosis of Caco-2 cells. Our results extend the reparative effect of OCA on impaired intestinal barrier function; however, we only detected the expression of FXR-related genes, the mechanism by which the FXR promoted intestinal epithelial proliferation and inhibited apoptotic cell death warrants further study.

Microbiota and intestinal mucosal barrier are mutually influenced. Normal microbiota applies trophic effects on the intestinal mucosa, which display a significant role in mucosal protection and epithelial regeneration. Recently, the role of microbiota in maintaining the intestinal barrier has increasingly attracted more research attention. Probiotics and fecal microbiota transplantation are widely used in intestinal inflammatory diseases (Al-Sadi et al., 2021; Dou et al., 2021). The PICRUST2 analysis showed no difference in functional microbiota profiles of the LPS biosynthesis pathway among the three groups. These results implied that the elevation of plasma LPS in the BDL group and BA patients were primarily attributable to intestinal barrier dysfunction and microbiota dysbiosis, which has been associated with an increase in the levels of Gram-negative microbiota. Our results suggest that increased LPS produced by intestinal dysbiosis might damage intestinal barrier function, in which OCA treatment improved the intestinal barrier in biliary obstruction by modulating microbiota. In response to OCA, the increased bacterial load was normalized and intestinal dysbiosis improved in BDL rats, which showed that the beneficial effects of OCA on intestinal epithelial apoptosis in biliary obstruction are likely due to remodeling of intestinal microbiota. BSH is a principal enzyme that stimulates the “gateway” reaction in the bacterial metabolism of conjugated bile acid to produce deconjugated bile acid. OCA treatment rectifies the rising relative abundance of Bacteroidetes and the decreased relative richness of Firmicutes at the phylum level, which increases enrichment of the gut microbiota with BSH-containing phyla. Furthermore, *Lactobacillus* plays a key role in maintaining the intestinal mucosal barrier by increasing immunoglobulin secretion (Liu et al., 2021; Niu et al., 2021; Yang et al., 2021; Zhang et al., 2021). Our results showed that OCA treatment exhibited more abundance of *Lactobacillus*, thereby protecting against an impaired intestinal mucosal barrier in obstructive jaundice.

Intestinal microbiota displays various potential phenotypes and functions in response to changes in a different environment. Against colonization of potentially harmful bacteria in the intestine is a major function for normal intestinal microbiota (Dai and Walker, 1999). We observed that the OCA treatment significantly decreased the high abundance of potentially pathogenic and gram-negative microflora in the BDL group, mostly attributed to the change in the abundance of Bacteroidetes. LPS derived from gram-negative bacteria can cause inflammatory responses and liver injuries (Rietschel et al., 1994; Machida et al., 2006; Aloman et al., 2007). This result is consistent with the level of LPS and TNF-α in the current study. In addition, analysis of KEGG pathway enrichment showed that OCA treatment greatly influences genes belonging to lipid metabolism pathways, including steroid hormone biosynthesis, sphingolipid metabolism, and primary and secondary bile acid biosynthesis.

Furthermore, it was concluded that the changed pathways in lipid metabolism were associated with biliary obstruction and OCA treatment. Biliary obstruction decreased bile acid metabolism-related genes, which were thought to be involved with inhibition of the FXR pathway. OCA treatment greatly stimulated activity of the FXR pathway, increasing the expression of bile acid metabolism-related genes. Recent studies have shown that intestinal FXR plays an indispensable role in glycolipid metabolism regulation, and intestinal-specific FXR agonists or antagonists participate in glucose and lipid metabolism regulation *in vivo* (Jiang et al., 2015a; Jiang et al., 2015b; Grundy, 2016). Our results also demonstrated that OCA attenuated the relative abundances of the genes involved in carbohydrate metabolism in biliary obstruction. The results of KEGG metabolic pathway analysis suggested that OCA had beneficial effects on BDL rats, which may be closely related to effective regulation of microbial metabolic pathways.

In general, OCA improved mucosal barrier function, down-regulated the concentrations of TNF-α and LPS, decreased the richness and diversity of the gut microbiota in the ileum, and reversed metabolic disorders.

## Conclusion

Our study indicated that the active FXR pathway by OCA had beneficial effects on the intestinal barrier in BDL rats. Specifically, OCA changed the composition and structure of the intestinal microbiota and improved the metabolic function of the intestinal microbiota by increasing the relative abundance of beneficial bacteria and reducing the relative abundance of harmful bacteria. Moreover, OCA promoted the recovery of the intestinal barrier function of BDL rats by downregulating the levels of inflammatory cytokines and LPS. Therefore, this research provides a theoretical and application basis for developing the FXR pathway as a functional target to alleviate intestinal barrier injury in biliary obstruction.

## Data Availability

The data presented in the study are deposited in the SRA of the NCBI (https://www.ncbi.nlm.nih.gov/sra), accession number SRR18791516-SRR18791534.

## Abbreviations

FXR, farnesoid X receptor; OCA, Obeticholic acid; ALT, alanine aminotransferase; AST, aspartate aminotransferase; ALP, alkaline phosphatase; BDL, bile duct ligation; TNF-α, tumor necrosis factor-alpha; FGF15/19, fibroblast growth factor 15/19; SHP, small heterodimer partner; LPS, lipopolysaccharide; SHP, small heterodimer partner; PCNA, Proliferating Cell Nuclear Antigen; T-βMCA, taurine-β-muricholic acid; ASBT, Apical Sodium-BA transporter; I-BABP, Induction of intestinal BA-binding protein; OSTα, Organic Solute and Steroid Transporter Alpha; CYP7A1, Cholesterol 7-alpha Hydroxy-lase.
