# Supplementary material for: Effects of Intestinal FXR-Related Molecules on Intestinal Mucosal Barriers in Biliary Tract Obstruction
Source: Front Pharmacol. 2022 Jun 13;13:906452. doi: 10.3389/fphar.2022.906452 (PMC9234329; doi:10.3389/fphar.2022.906452)
Supplement: Supplementary file 1 [file DataSheet1.DOCX]

**Supplementary Figure**

**Treatment with OCA reduced liver fibrosis in BDL rats**

We determined if OCA reduced liver fibrosis using three methods. In BDL rats, hepatocellular edema, bile duct hyperplasia (red arrows), and fibrosis tissue formation (black arrows) were clearly visible. Bile duct hyperplasia and fibrosis tissue production were significantly reduced by OCA (Figure S1A). Stimulation of FXR by OCA significantly attenuated liver fibrosis in BDL rats. The percentage of Sirius red- and Masson-stained areas in BDL rats was significantly higher than that in controls, which was improved by OCA (Figure S1B, C). Moreover, the serum ALT and AST aried according to degree of liver injury (Figure S1D, E).


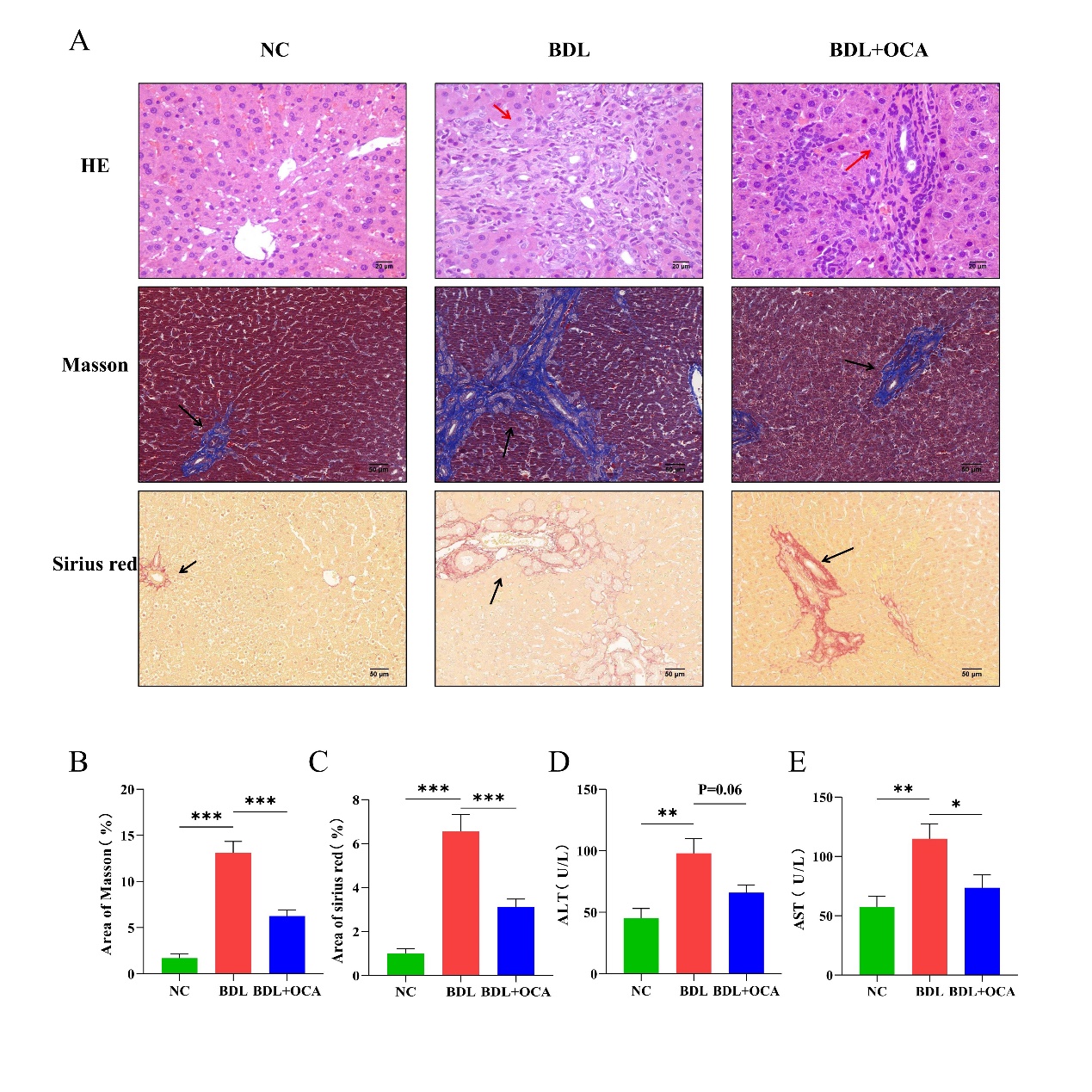


Figure S1. Treatment with OCA reduces liver fibrosis in BDL rats. (A) Representative images of liver specimens stained with hematoxylin and eosin, Sirius red, and Masson. (B, C) Fibrosis was quantified by morphometric measurements of Masson and Sirius red. (D, E) Serum levels of ALT and AST activities. The data are expressed as the mean ± SEM (n = 7-9). (**P* < 0.05, ***P* < 0.01, ****P* < 0.001).
